# Safety and efficacy of immediate heparin reversal with protamine after complex percutaneous coronary intervention

**DOI:** 10.1186/s12872-022-02650-5

**Published:** 2022-05-10

**Authors:** Jin Hee Choi, Kook Jin Chun, Soon Myung Jung, Soo Yong Lee, Min Ku Chon, Sang Hyun Lee, Ki Won Hwang, Jeong Su Kim, Yong-Hyun Park, June Hong Kim

**Affiliations:** Division of Cardiology, Department of Internal Medicine, Pusan National University Yangsan Hospital, 20, Geumo-ro, Mulgeum-eup, Yangsan, Gyeongnam 626-770 South Korea

**Keywords:** Anticoagulant, Bleeding, Percutaneous coronary intervention, Protamine

## Abstract

**Background:**

Compared to simple percutaneous coronary intervention (PCI), complex PCI is associated with higher bleeding and thrombotic risk. No previous study has evaluated the use of protamine after PCI with contemporary technologies. This study aimed to evaluate the safety and efficacy of manual compression with and without protamine after transfemoral complex PCI.

**Methods:**

We retrospectively analyzed 160 patients (protamine group, n = 92; non-protamine group, n = 68) who underwent complex PCI via the femoral artery. The primary outcome was a composite of in-hospital death, myocardial infarction, stent thrombosis, stroke/systemic embolism, bleeding requiring blood transfusion, and vascular access complications.

**Results:**

The primary outcome was significantly lower in the protamine group than in the non-protamine group (4.3% vs. 17.6%; *p* = 0.006). This was driven mainly by the lower incidences of hematoma in the protamine group (3.3% vs. 13.2%, *p* = 0.020). Furthermore, the protamine group had a significantly shorter hospital stay than the non-protamine group (4.8 ± 3.7 days vs. 8.4 ± 8.3 days, *p* = 0.001). While > 90% of the patients had acute coronary syndrome, there were no incidences of myocardial infarction or stent thrombosis in either group.

**Conclusions:**

Among patients who underwent complex PCI via transfemoral access, immediate protamine administration was associated with a significantly lower rate of vascular access complications, especially hematoma, and shorter hospital stay than no protamine administration.

## Background

Complex percutaneous coronary intervention (PCI) is likely to increase the risk of bleeding complications due to the use of larger French sheath systems via a transfemoral approach, dual access, and a large volume of heparin [1, 2]. Therefore, complete hemostasis after femoral sheath removal is the most crucial factor. Although vascular closure devices can be used to achieve early hemostasis and ambulation compared to manual compression, these devices still have rare but serious complications, including groin infection, distal ischemia, and localized thrombosis [3]. Furthermore, the calcified or small size of the femoral artery has some limitations in using the vascular closure devices, which eventually require manual compression in challenging cases [4]. Patients with successful complex PCI remain at increased risk of bleeding and stent thrombosis due to incomplete stent expansion, bifurcation stenting, and use of multiple long stents. Several studies have shown that immediate anticoagulant reversal with protamine and sheath removal is a safe alternative to standard manual compression after PCI [5–7]. As complex PCI itself has a higher thrombotic risk than simple PCI, the use of protamine is expected to increase the risk of stent thrombosis. To our knowledge, no previous study has evaluated the immediate use of protamine after complex PCI using contemporary technologies. Therefore, we evaluated the safety and efficacy of transfemoral PCI in patients with complex coronary lesions using manual compression with or without protamine.

## Methods

From January 2015 to December 2019, 3976 consecutive patients (7007 lesions) underwent PCI at the Pusan National University Yangsan Hospital. The inclusion criteria were (1) femoral artery puncture requiring a > 7-Fr guiding system; (2) complex PCI, which was defined as a procedure with at least one of the following angiographic characteristics: unprotected left main disease, ≥ 3 lesions treated, lesion length ≥ 60 mm, bifurcation treated with two-stent technique, or chronic total occlusion (CTO) lesions as the target lesion; and (3) manual compression, which was not suitable for arterial closure devices such as Perclose Proglide (Abbott Vascular Devices, Redwood City, CA, USA) and Angio-Seal (Terumo Medical Corporation, Somerset, NJ, USA) because of access site locations, dense calcifications, and small size of the femoral artery. The exclusion criteria were as follows: (1) use of oral anticoagulants, (2) presence of an intracoronary thrombus, and (3) hemodynamic instability. In this study, only the first PCI was considered for analysis for patients who underwent multiple PCI. A total of 160 patients who met the inclusion and exclusion criteria were retrospectively analyzed. The study population was divided into patients who received protamine (protamine group) and those who did not receive protamine (non-protamine group). This study was approved by the Pusan National University Yangsan Hospital Institutional Review Board (IRB No. 05-2020-105), and the requirement for written informed consent was waived because of the retrospective study design.

The baseline clinical and procedural data were retrospectively collected. The primary outcome was a composite of in-hospital death, myocardial infarction (MI), stent thrombosis, stroke/systemic embolism (SSE), bleeding requiring blood transfusion, and vascular access complications. Vascular access complications were defined as the presence of a major groin hematoma (> 5 cm in diameter), pseudoaneurysm, arteriovenous fistula, or surgical repair. Protamine-related adverse effects included hypotension, bradycardia, anaphylactic reactions, and pulmonary hypertension.

Before the procedure, all patients received dual antiplatelet therapy (DAPT), including clopidogrel (loading dose, 600 mg; maintenance dose, 75 mg once daily), ticagrelor (loading dose 180 mg, maintenance dose 75 mg twice daily), or prasugrel (loading dose 60 mg, maintenance dose 10 mg once daily) with acetylsalicylic acid (loading dose 300 mg, maintenance dose 100 mg daily). PCI was performed using standard techniques. Unfractionated heparin was administered as an initial bolus of 70–100 UI/kg, and additional boluses were administered during the procedure to achieve an activated clotting time (ACT) of 250–300 s. The choice of stent type and device was left to the discretion of the interventional cardiologist. Intravascular ultrasound (IVUS)-guided PCI was performed in all patients. A successful angiographic procedure was defined as residual stenosis < 30%, TIMI grade 3 distal flow, and absence of significant dissection. At the end of PCI, the ACT was checked. The use of protamine was maintained at the physician’s discretion. If ACT was < 200 s, no protamine was administered. If ACT was ≥ 200 s, 25–50 mg of protamine (diluted in 100 mL of 0.9% normal saline) was administered intravenously for 10 min. The protamine injection rate was maintained at ≤10 mg/min to avoid hypotension and pulmonary edema [8, 9]. In the protamine group, the sheath was removed immediately after protamine administration. In the non-protamine group, the sheath was generally removed 2–3 h after PCI. Hemostasis was achieved using manual compression or mechanical compression devices, such as a C-shaped clamp. After compression, all patients were instructed to take absolute bed rest for 4–5 h, with a sandbag placed on the puncture site.

### Statistical analysis

All continuous variables are expressed as the mean ± standard deviation. Normally and non-normally distributed continuous variables were compared using the Student’s t-test and Mann–Whitney U test, respectively. Categorical variables were expressed as absolute numbers (frequency) and percentages and compared using Pearson’s chi-square test or Fisher’s exact test. Independent predictors of the primary outcome were identified by first including the parameters in a univariate regression analysis and subsequently entering the significant predictors in a stepwise multivariate logistic regression model.

Statistical significance was defined as a two-tailed *p* value of ≤ 0.05. Statistical analyses were performed using SPSS version 18.0 for Windows (IBM Corp., Armonk, NY, USA).

## Results

A total of 160 patients were enrolled and divided into the protamine group (n = 92) and the non-protamine group (n = 68). The baseline patient characteristics are shown in Table 1. The demographic and clinical characteristics were balanced between the two groups. The mean age was 68.6 ± 10.4 years and 67.5% of patients were male. Notably, more than 90% of the patients in both groups had acute coronary syndrome (ACS) (91.3% in the protamine group and 95.6% in the non-protamine group).

**Table 1:** Baseline demographic and clinical characteristics of patients in the protamine and non-protamine groups

	All (n = 160)	Protamine group (n = 92)	Non-protamine group (n = 68)	*p* value
Age, years	68.6 ± 10.4	68.1 ± 10.9	69.2 ± 9.7	0.518
Male sex	108 (67.5)	61 (66.3)	47 (69.1)	0.707
Body mass index, kg/m^2^	24.2 ± 3.4	24.4 ± 3.2	23.9 ± 3.5	0.329
Smoking	43 (26.9)	27 (29.3)	16 (23.5)	0.412
Cardiac or coexisting conditions				
Hypertension	103 (64.4)	57 (62.0)	46 (67.6)	0.457
Diabetes mellitus	76 (47.5)	38 (41.3)	38 (55.9)	0.068
Chronic kidney disease	31 (19.4)	15 (16.3)	16 (23.5)	0.253
Previous myocardial infarction	18 (11.3)	12 (13.0)	6 (8.8)	0.404
Previous PCI	32 (20.0)	20 (21.7)	12 (17.6)	0.522
Previous CABG	7 (4.4)	5 (5.4)	2 (2.9)	0.700
Dyslipidemia	79 (49.4)	44 (47.8)	35 (51.5)	0.649
Hemoglobin, g/dL	12.1 ± 2.4	12.0 ± 2.5	12.1 ± 2.2	0.783
Platelet, × 10^3^ cells/mL	219.1 ± 64.8	219.9 ± 61.3	218.0 ± 69.7	0.855
Left ventricular ejection fraction, %	54.3 ± 12.5	54.6 ± 12.4	53.9 ± 12.7	0.721
Clinical presentation				0.357
Stable CAD	11 (6.9)	8 (8.7)	3 (4.4)	
ACS*	149 (93.1)	84 (91.3)	65 (95.6)	
Dual antiplatelet therapy				0.069
Aspirin and clopidogrel	97 (60.6)	58 (63.0)	39 (57.4)	
Aspirin and ticagrelor	46 (28.8)	21 (22.8)	25 (36.8)	
Aspirin and prasugrel	17 (10.6)	13 (14.1)	4 (5.9)	

Values are n (%) or mean ± standard deviation

*ACS* acute coronary syndrome, *CABG* coronary artery bypass grafting, *CAD* coronary artery disease, *PCI* percutaneous coronary intervention

*Includes unstable angina, non-ST-segment elevation myocardial infarction, or ST-segment elevation myocardial infarction

The angiographic and procedural characteristics of the two groups are shown in Table 2. The non-protamine group had more unprotected left main disease cases than the protamine group (29.4% vs. 17.4%, *p* = 0.072). There were significantly more cases of CTO as a target lesion treated in the protamine group than in the non-protamine group (62.0% vs. 30.9%, *p* < 0.001). All patients underwent IVUS-guided PCI using second-generation drug-eluting stents (DES).

**Table 2:** Angiographic and procedural characteristics of patients in the protamine and non-protamine groups

	All (n = 160)	Protamine group (n = 92)	Non-protamine group (n = 68)	*p* value
Multivessel disease	119 (74.4)	67 (72.8)	52 (76.5)	0.602
Lesion type B2/C	131 (81.9)	77 (83.7)	54 (79.4)	0.487
Number of stents (per patient)	2.4 ± 1.2	2.4 ± 1.3	2.4 ± 1.1	0.963
Treated lesion				
Unprotected left main disease	36 (22.5)	16 (17.4)	20 (29.4)	0.072
≥ 3 lesions	26 (16.3)	15 (16.3)	11 (16.2)	0.983
Lesion length ≥ 60 mm	90 (56.3)	52 (56.5)	38 (55.9)	0.936
Bifurcation treated with two-stent technique	33 (20.6)	19 (19.6)	14 (20.6)	0.873
Chronic total occlusion	78 (48.8)	57 (62.0)	21 (30.9)	< 0.001
Stent				
Total length, mm	68.7 ± 39.2	68.9 ± 41.5	68.4 ± 36.1	0.930
Minimal diameter, mm	2.89 ± 0.42	2.89 ± 0.42	2.89 ± 0.41	0.975
Drug eluting stent				
Everolimus	98 (61.3)	58 (63.0)	40 (58.8)	0.588
Zotarlimus	49 (30.6)	29 (31.5)	20 (29.4)	0.775
Sirolimus	15 (9.4)	12 (13.0)	3 (4.4)	0.098
Biolimus	7 (4.4)	5 (5.4)	2 (2.9)	0.700
Use of IVUS	160 (100)	92 (100)	68 (100)	–
Sheath diameter				
7 Fr	144 (90.0)	80 (87.0)	64 (94.1)	0.219
8 Fr	26 (16.3)	18 (19.6)	8 (11.8)	0.186
Heparin dose, unit	8762.5 ± 1835.1	8788.0 ± 1751.3	8727.9 ± 1955.5	0.838
ACT at the end of PCI, seconds	316.8 ± 73.7	320.2 ± 76.0	293.9 ± 53.0	0.252
Protamine dose, mg	–	45.9 ± 8.0	–	–
Procedural time, minutes	104.4 ± 56.1	108.2 ± 55.5	99.2 ± 57.0	0.317

Values are n (%) or mean ± standard deviation

*ACT* activated clotting time, *IVUS* intravascular ultrasound, *PCI* percutaneous coronary intervention

**Fig. 1:**
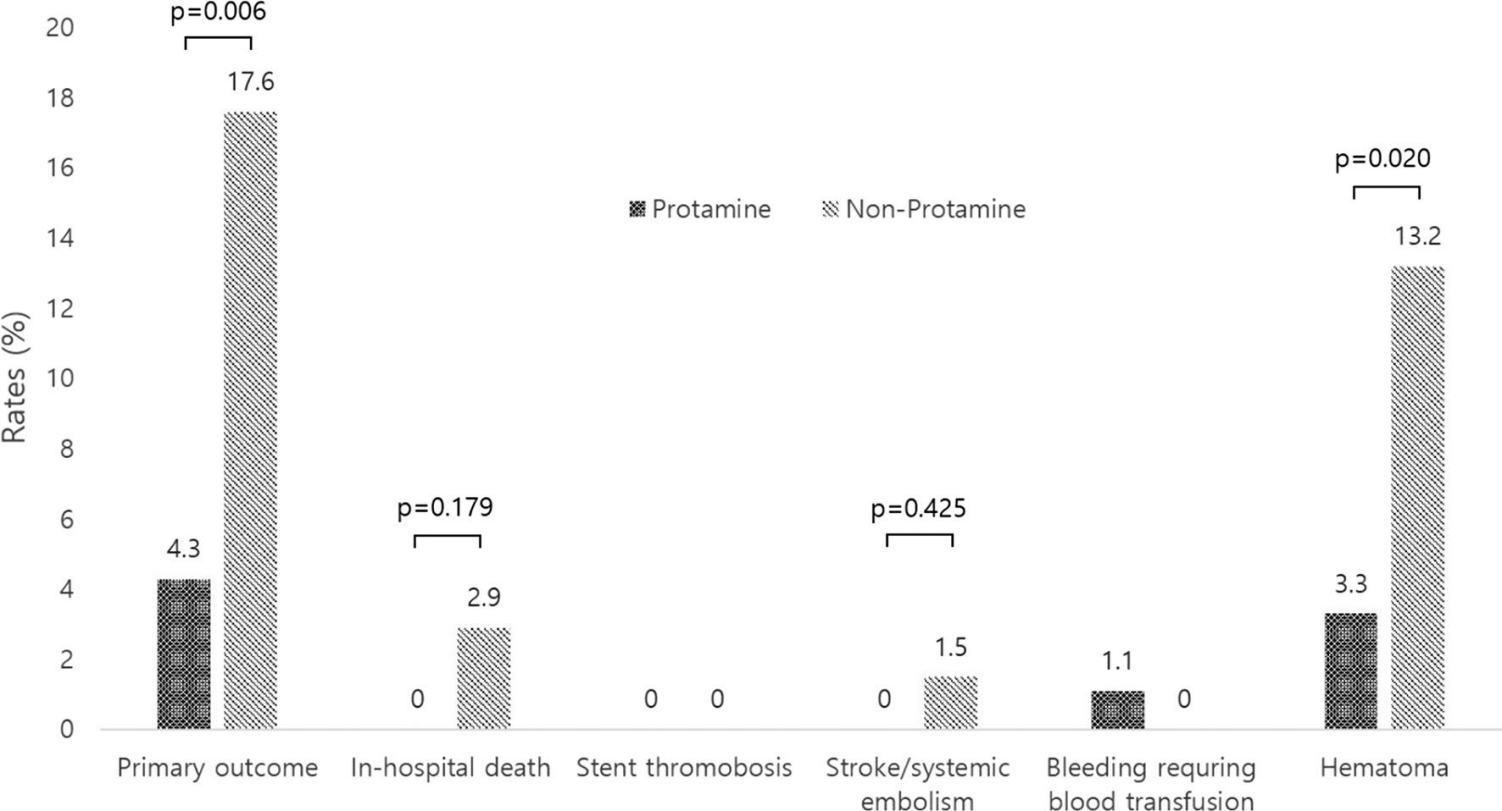
Clinical outcomes of the patients according to protamine administration. Primary outcome = a composite of in-hospital death, myocardial infarction, stent thrombosis, stroke/systemic embolism, bleeding requiring blood transfusion, and vascular access complications

The primary outcome was significantly lower in the protamine group than in the non-protamine group (4.3% vs. 17.6%, *p* = 0.006) (Table 3, Fig. 1). The significantly lower rates in the protamine group were driven mainly by lower rates of hematoma in the protamine group than in the control group (3.3% vs. 13.2%, *p* = 0.020). No stent thrombosis or MI was observed in either group. There were no adverse reactions related to protamine, such as hypotension, bradycardia, anaphylactic reaction, bronchospasm, and pulmonary hypertension. In addition, the protamine group had a significantly shorter length of in-hospital stay than the non-protamine group (4.8 ± 3.7 vs. 8.4 ± 8.3 days, *p* = 0.001).

**Table 3:** Clinical outcomes in the protamine and non-protamine groups

	All (n = 160)	Protamine group (n = 92)	Non-protamine group (n = 68)	*p* value
Primary outcome	16 (10.0)	4 (4.3)	12 (17.6)	0.006
In-hospital death	2 (1.3)	0 (0.0)	2 (2.9)	0.179
Myocardial infarction	0 (0.0)	0 (0.0)	0 (0.0)	–
Stent thrombosis	0 (0.0)	0 (0.0)	0 (0.0)	–
Stroke/systemic embolism	1 (0.6)	0 (0.0)	1 (1.5)	0.425
Bleeding requiring blood transfusion	1 (0.6)	1 (1.1)	0 (0.0)	0.575
Vascular access complications				
Hematoma	12 (7.5)	3 (3.3)	9 (13.2)	0.020
Pseudoaneurysm	0 (0.0)	0 (0.0)	0 (0.0)	–
Arteriovenous fistula	0 (0.0)	0 (0.0)	0 (0.0)	–
Surgical repair	0 (0.0)	0 (0.0)	0 (0.0)	–
In-hospital length of stay, (days)	6.3 ± 6.3	4.8 ± 3.7	8.4 ± 8.3	0.001

Values are n (%) or mean ± standard deviation

**Fig. 2:**
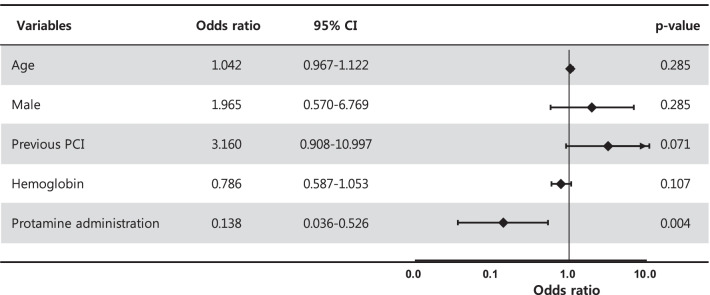
Forest plot of multivariate logistic regression analysis for predictors associated with the primary outcome. *CI* confidence interval, *PCI* percutaneous coronary intervention

Univariate and multivariate analyses revealed that only the administration of protamine (odds ratio, 0.138; 95% confidence interval: 0.036–0.526; *p* = 0.004) was independently associated with the primary endpoint (Fig. 2).

## Discussion

The main findings of our study, with a total of 160 patients undergoing transfemoral PCI for complex lesions, are as follows: (1) immediate administration of protamine resulted in significantly lower rates of the composite of in-hospital death, MI, stent thrombosis, stroke/systemic embolism, bleeding requiring blood transfusion, and vascular access complications; (2) use of protamine after PCI was safe with lower rates of hematoma and without increasing any stent thrombosis and MI; and (3) patients with protamine administration had significantly shorter hospital stays than patients without protamine.

Although the incidence of bleeding complications after PCI has been decreasing, it remains the main challenge in complex PCI, which requires a femoral approach, larger sheath size, and more prolonged ACT. Stent thrombosis is a rare but serious complication of complex PCI due to procedure-related factors, such as bifurcation, long calcified lesions, and CTO [10].

Protamine has been used clinically for prompt reversal of the anticoagulant effect of heparin. Although protamine has been widely used in cardiovascular surgery [11] for a long time, it is underused for PCI because of the possible increase in the chances of hyperacute stent thrombosis, heparin rebound, and the potential for allergic or anaphylactic reactions [12]. Nevertheless, some studies have investigated the safety of protamine use following PCI. De Luca et al. [5] performed a meta-analysis of randomized and non-randomized trials from 1990 to 2009 to evaluate the safety and benefits of protamine administration after coronary angiography, including bare-metal stents and first-generation DES. In this meta-analysis of 6762 patients, the rates of short-term mortality and MI were similar in both groups, with a significant reduction in major bleeding complications in patients receiving protamine. Yamamoto et al. [6] showed the safety of protamine following elective transfemoral PCI with the second-generation DES. They showed that the use of protamine after manual compression following elective transfemoral PCI was associated with fewer bleeding complications and protamine-treated patients did not sustain higher rates of stent thrombosis than non-protamine-treated patients, despite using DES. However, Yamamoto et al. [6] excluded patients with ACS who had a higher risk of bleeding and thrombosis than those with stable angina and did not describe the complexity of the lesion. In our study, we included patients who underwent only complex PCI, and more than 90% of the patients had ACS. Our study suggests that the administration of protamine is safe and does not increase the risk of stent thrombosis or MI.

Despite the inclusion of patients who underwent complex PCI with high anatomical risk, the reasons for the absence of stent thrombosis events in the protamine group should be considered. First, at the time of PCI, we only included patients who were already treated with DAPT, including new-generation P2Y12 inhibitors such as ticagrelor or prasugrel. DAPT has been established as a standard-of-care treatment for preventing stent- and non-stent-related ischemic events after PCI with DES [13–15]. Stent thrombosis appears to be significantly affected by the potency and rapidity of antiplatelet therapy, and the lack or delayed effect of antiplatelet agents has consistently been associated with a higher risk of stent thrombosis [16]. Compared with clopidogrel, ticagrelor and prasugrel, which have greater potency and faster action in inhibiting adenosine diphosphate–induced platelet aggregation; thus, they can reduce stent thrombosis regardless of stent type, the timing of stent thrombosis, and ACS [17, 18]. Second, all patients in our study used second-generation DES, which has a lower rate of stent thrombosis than first-generation DES. First-generation DES platforms, which have relatively thick struts, durable polymer coating that can cause peri-strut inflammation, and paclitaxel that may cause delayed endothelial recovery, were associated with late and very late stent thrombosis [19, 20]. However, second-generation DES platforms have lower thrombogenicity due to more flexible and thinner struts, more biocompatible or biodegradable polymers, and limus drugs decrease neointimal response and increase re-endothelialization [21, 22]. Third, IVUS-guided PCI was performed in all patients in our study. PCI for complex lesions such as small-vessel disease, bifurcation, and long or highly calcified lesions is associated with a higher risk of malapposition, incomplete lesion coverage, under-expansion, and the likelihood of a slower or non-uniform pattern of endothelialization compared with simple PCI. In our study, optimal PCI with proper stent sizing and stent deployment using pre-intervention and post-intervention IVUS contributed to the reduction of stent thrombosis by aiming at no residual narrowing, absence of dissections, complete stent expansion, and good stent apposition [10, 23, 24].

This study had several limitations that should be addressed. This was a retrospective, non-randomized, single-center study. However, to the best of our knowledge, this is the first study to evaluate patients receiving heparin reversal with protamine for complex PCI. In addition, the relatively small study population may have affected the outcome. The cumulative incidence of stent thrombosis with DES at one year was very low at less than 1% [24]; therefore, the incidence of stent thrombosis may have been underestimated due to the small number of patients in this study. Anaphylactic reactions to protamine may also have been underestimated due to the small study population because they were very rare (< 1%) and less likely to occur without protamine-containing insulin [25].

## Conclusions

Among patients undergoing complex PCI via transfemoral access, immediate protamine use with manual compression after PCI resulted in significantly lower rates of the primary endpoint, driven mainly by significantly lower rates of vascular access complications, especially hematoma. In addition, protamine administration after complex PCI significantly shortened the hospital stay. Further prospective studies are needed to validate the safety and efficacy of protamine following complex PCI.

## Data Availability

The datasets generated and/or analyzed during the current study are not publicly available due to privacy or ethical restrictions but are available from the corresponding author on reasonable request.

## Abbreviations

PCI: Percutaneous coronary intervention
CTO: Chronic total occlusion
MI: Myocardial infarction
DAPT: Dual antiplatelet therapy
ACT: Activated clotting time
IVUS: Intravascular ultrasound
ACS: Acute coronary syndrome
DES: Drug-eluting stents
